# An important step towards a prevascularized islet macroencapsulation device—effect of micropatterned membranes on development of endothelial cell network

**DOI:** 10.1007/s10856-018-6102-0

**Published:** 2018-06-25

**Authors:** Katarzyna Skrzypek, Milou Groot Nibbelink, Lisanne P. Karbaat, Marcel Karperien, Aart van Apeldoorn, Dimitrios Stamatialis

**Affiliations:** 1 Bioartificial organs, Biomaterials Science and Technology, MIRA Institute of Biomedical Technology and Technical Medicine, University of Twente, Enschede, The Netherlands; 2 Developmental BioEngineering, MIRA Institute of Biomedical Technology and Technical Medicine, University of Twente, Enschede, The Netherlands; 3 Present Address: Complex Tissue Regeneration, MERLN Institute for Technology Inspired Regenerative Medicine, Maastricht University, Maastricht, The Netherlands

## Abstract

The development of immune protective islet encapsulation devices could allow for islet transplantation in the absence of immunosuppression. However, the immune protective membrane / barrier introduced there could also impose limitations in transport of oxygen and nutrients to the encapsulated cells resulting to limited islet viability. In the last years, it is well understood that achieving prevascularization of the device in vitro could facilitate its connection to the host vasculature after implantation, and therefore could provide sufficient blood supply and oxygenation to the encapsulated islets. However, the microvascular networks created in vitro need to mimic well the highly organized vasculature of the native tissue. In earlier study, we developed a functional macroencapsulation device consisting of two polyethersulfone/polyvinylpyrrolidone (PES/PVP) membranes, where a bottom microwell membrane provides good separation of encapsulated islets and the top flat membrane acts as a lid. In this work, we investigate the possibility of creating early microvascular networks on the lid of this device by combining novel membrane microfabrication with co-culture of human umbilical vein endothelial cell (HUVEC) and fibroblasts. We create thin porous microstructured PES/PVP membranes with solid and intermittent line-patterns and investigate the effect of cell alignment and cell interconnectivity as a first step towards the development of a stable prevascularized layer in vitro. Our results show that, in contrast to non-patterned membranes where HUVECs form unorganized HUVEC branch-like structures, for the micropatterned membranes, we can achieve cell alignment and the co-culture of HUVECs on a monolayer of fibroblasts attached on the membranes with intermittent line-pattern allows for the creation of HUVEC branch-like structures over the membrane surface. This important step towards creating early microvascular networks was achieved without the addition of hydrogels, often used in angiogenesis assays, as gels could block the pores of the membrane and limit the transport properties of the islet encapsulation device.

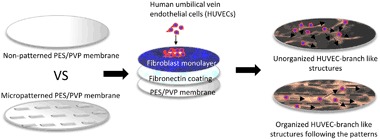

## Introduction

Over the last decade, improvements in islet isolation techniques have made islet transplantation an option for treatment of certain groups of patients with type 1 diabetes. Although islet transplants have shown improved graft function, the patients still require immunosuppression to prevent rejection [1]. Islet encapsulation using semi-permeable membranes could offer a solution to avoid the need for toxic immunosuppression while increasing the chances of graft survival and function. The primary role of encapsulation is to create a barrier against immune cells and cytotoxic molecules, thus avoid rejection while still allowing for the diffusion of oxygen, nutrients and hormones [2].

Islets are highly metabolic and require high amounts of oxygen and glucose to function properly. In fact, they are highly vascularized and receive up to 5-15% of pancreatic blood flow while they account for only 1-2% of the entire pancreas mass [3]. Islet capillary network in combination with a high islet blood-flow rate ensures that cells receive oxygen and nutrients at near arterial levels [4]. Unfortunately, the isolation procedure disrupts their own vasculature, thus, immediately following transplantation, islets face hypoxic stress that contributes to a loss of 60% of transplanted islets during the first 48 h post-transplantation [5]. In order to provide sufficient oxygen to mitochondria inside a cell, the maximal distance between capillary and cell must not exceed 200 µm [2]. Therefore, a very important consideration in the development of a bioartificial pancreas is the transplantation site where the encapsulated islets are in close proximity to the blood stream [6]. Due to the significantly greater graft volume, the geometry and the material, encapsulated islets cannot be safely transplanted into the liver through the portal system [7]. Therefore, other sites like subcutaneously, the omental pouch, bone marrow or the peritoneal cavity have been proposed as well [1]. Despite each site’s distinctive advantages, the significant graft failure is attributed to lack of adequate oxygenation. Oxygen supply to encapsulated islets is highly complex and depends on several factors e.g. the oxygen partial pressure (pO_2_) in the blood at the implantation site, distribution of host blood vessels in the vicinity of the implant surface as well as spatial arrangement of the encapsulated cells. In order to maintain full functionality of all islet cells, the pO_2_ level should be about 45-50 mmHg [8]. One of the approaches to enhance oxygen supply is the induction of neovascularization adjacent to the immunoisolation device in order to bring the blood flow close to the tissue [2, 8].

Currently, various strategies are under investigation to improve islet vascularization, including the addition of growth factors to induce a faster vascularization rate and prevascularization of the device [9, 10]. The application of angiogenic growth factors such as vascular endothelial growth factor, nerve growth factor and basic fibroblast growth factor has shown to improve graft functionality by increasing angiogenesis locally [11]. However, the complete vascularization of large implants by angiogenesis still needs a prolonged time period while hypoxic conditions contribute to a large tissue loss [12]. Moreover, inappropriate administration of growth factors can also result in abnormal and unsuitable vasculature formation [13]. Prevascularization of the encapsulation device can be obtained directly in vivo, where a non-vascularized construct is implanted a few days/weeks before the islets are seeded in a highly vascularized area [9]. In fact, Serenova Corporate proposed a biocompatible macrodevice where non-biodegradable knitted polymer mesh with large pores allows for the development of fibrous tissue rich in blood vessels [1]. Inside the device, a rod-like polymer plug is positioned to guide the growth of the micro-vessels and connective tissue in order to create lumen for the future transplantation of islets. When the lumen is created, the rod-like plug is removed and islets are transplanted. However, this approach requires advanced imaging technics in order to optimize the time period required for the development of vasculature after which islets can be implanted [14].

Another islet macroencapsulation device designed to be incorporated into the host vasculature is the TheraCyte^***TM***^ system [15]. The device is composed of two thin polytetrafluoroethylene membranes. The outer membrane with pores of 5 μm improves the strength of the device and allows for infiltration of vasculature, whereas the additional inner 0.4 μm pore membrane provides islet immunoisolation. Although, the device is well vascularized, it requires several months to provide optimal blood perfusion in the surrounding microcirculation and therefore glucose and insulin diffusion is impaired during the first period after transplantation. Nonetheless, it has been shown that the preimplantation can improve vascularization of immune protective devices before islet transplantation [16]. Although this seems to be a promising strategy, it requires several surgical steps that could be reduced by induced prevascularization in vitro. Here, after prevascularized structures are formed, islets can be encapsulated and the final construct is implanted to the patient. This method is time saving as the blood vessels from the host do not have to infiltrate through the construct but only connect to the pre-existing network [17].

The most widely applied in vitro prevascularization approach is the seeding of vessel-forming cells onto scaffolds [12]. The main cell type in the native vasculature is the endothelial cell. However, years of research on angiogenesis has shown that co-culture systems of multiple cell types are required to have the advantage of cell-cell contact and cross-talk between the different cell types [18]. In fact, co-cultures with fibroblasts, mesenchymal stem cells and smooth muscle cells have shown promising results in the development of capillary like structures in vitro [19–21]. However, during in vitro generation of microvascular networks it is important to achieve close representation of native tissues, which are highly organized at the microscale level [22]. Here, the construct microarchitecture has a potential to support and guide prevascular network formation. Often, physical guides (e.g. wells, channels) have been applied to create cell patterns [23]. This approach can be used to enhance 2-dimensional (2D) endothelial cells alignment and organization to promote 3-dimensional (3D) vasculature formation in tissue engineering constructs.

In our previous study, we developed a functional flat macroencapsulation device consisting of two polyethersulfone/polyvinylpyrrolidone (PES/PVP) membranes, where a bottom microwell membrane provides good separation of encapsulated islets and the top flat membrane acts as a lid [24]. In order to reduce the time required for the device to be incorporated within host vasculature and therefore provide proper islet oxygenation after transplantation, the outer membrane surface of our device could be used for the creation of a prevascularized layer in vitro (Supplementary figure 1).

In this work, we hypothesize that the creation of early microvascular networks on the lid of the device can be achieved by combining novel membrane microfabrication with co-culture of human umbilical vein endothelial cell (HUVEC) and human dermal fibroblasts (NHDFs). We, in fact, investigate the effect of the membrane surface micropattern on cell alignment and orientation which are important parameters for the development of a prevascularized network [25]. We aim to obtain this without the addition of hydrogels (e.g. matrigel) often used as angiogenesis assays [21], as these gels could block the membrane pores causing transport limitations and negatively affect islet function. Various surface topographies have been shown to affect cell morphology, differentiation and proliferation, which could be used to control cell growth and their orientation. The formation of capillary like structures has been obtained using grooves, stripes or adjacent fibers [26–28]. However, these methods did not result in interconnected capillary-like networks but in the formation of separated aligned endothelial cell structures and often required the addition of gels.

In this work, we prepare membranes with two patterns: one of intermittent lines and another with a combination of intermittent and solid lines (Fig. 2) with a distance of 100 µm between the patterns. We selected these patterns since earlier studies have shown that alignment occurs in channels as small as 20 µm up to 130 µm [29]. We hypothesize that these patterns would provide not only alignment but also interconnectivity of the cellular network, better mimicking the situation in the native tissues. Papenburg et al. have shown that such cell organization can be controlled using poly(L-lactic acid) membranes with continuous and interconnected microchannels [30].

The micropatterned PES/PVP membranes were fabricated via phase separation micromolding method (PSµM), which allows us to obtain porous membranes with desired surface topography in one step [30]. These membranes have low cell adhesion, which is favorable for islet encapsulation devices in order to maintain the cell native morphology [31]. Therefore, we apply a thin fibronectin coating on one side of the membrane to improve cell attachment properties. We study the attachment and alignment of normal human dermal fibroblasts (NHDFs) and human umbilical endothelial cells (HUVECs) on our micropatterned and non-patterned PES/PVP membranes. It has widely been demonstrated that fibroblasts promote endothelial cells proliferation, migration, and angiogenesis, both in vivo and in vitro [32, 33]. Therefore, we further use NHDFs as support cells in the co-culture with HUVECs and investigate whether the micropatterns have an effect on HUVEC structure formation and organization in comparison to the non-patterned membranes.

## Materials and methods

### Membrane preparation

A 15 wt% poly(ether sulfone) (PES, ULTRASON, the Netherlands), 5 wt% poly(vinylpyrrolidon) (PVP, 40.000 kDa, Sigma-Aldrich) polymer blend in N-methylpyrrolidone (NMP, Sigma-Aldrich) was used to fabricate porous membranes via PSµM [30]. The polymer blend was cast on a glass plate (for flat membranes) or on a custom made, micropatterned silicon wafer for the creation of the different micropatterns (Fig. 2b).

A casting thickness of 300 µm was used for all membrane fabrication. Directly after casting, the polymer was submerged in a coagulation bath containing demineralized water (dH_2_O). After the polymer solution became turbid and precipitated, the membranes were removed from the glass plate or micropatterned silicon wafer, and rinsed with dH_2_O to remove remaining solvent traces. For all experiments with cells, the membranes were plated in a 24-well plate and fixed with O-rings, after which they were sterilized (70% ethanol, 30 min) and washed with phosphate buffered saline (PBS, 3×). Membranes were washed 3× with dH_2_O before use.

### Membrane characterization

The membrane surface morphology and pore size were analyzed using scanning electron microscope (SEM). The membranes were dried overnight in air at room temperature. Dried membranes were placed on the SEM holders and sputter-coated with a nm-thick gold layer prior to imaging.

The membranes with an effective surface area of 0.9 cm^2^ were used for clean water flux measurements. The experiments were performed using nitrogen pressurized dead-end Amicon-type ultrafiltration cell and MiliQ water. Firstly, the membranes were pre-pressurized for at least 30 min at 0.6 bar. Afterwards, the clean water flux through the membrane at various transmembrane pressures was measured for at least 1 h. The membrane water permeability was calculated from the slope of the linear part of the flux versus the transmembrane pressure relation (*n* = 3).

Tensile tests, to determine the membrane tensile strength, were carried out at room temperature using a ZwickZ020 tensile tester (Germany) with a load cell of 500 N. The cross-head speed was 5 mm/min and the elongation was derived from grip-to-grip separation, which was initially 30 mm. The membrane samples measured approximately 10 by 0.5 by 0.03 cm. A set of six samples was analyzed and averaged. Young’s modulus, maximum tensile stress and elongation at break were calculated from the experimental stress–strain curves.

An optical contact angle device equipped with an electronic syringe unit (OCA15, Dataphysics, Germany) connected to a charge-coupled device (CCD) video camera was used for static water contact angle measurements of the membrane. A deionized water drop was deposited onto the membrane surfaces by the syringe, after which the drop contour was fitted by the Young-Laplace method. The contact angle of five different locations on the membrane surface was measured and the average values were reported as the contact angle for a membrane sample.

### Cell culture

Human umbilical vein endothelial cells (HUVEC, Lonza CC2519A) and normal human dermal fibroblasts (NHDF, ThermoFisher SCIENTIFIC C-013-5C) were purchased and upon arrival directly transferred to liquid nitrogen. Upon the start of a new culture, cells were grown to 80% confluence using Endothelial Growth Medium-2 (EGM-2, Lonza) and Fibroblast Growth Medium (FGM, Lonza). When 80% confluence was reached, cells were trypsinized. When the cells were completely detached from the cell culture flask, trypsin neutralizing medium (EGM-2 with 10% fetal bovine serum (FBS) or FGM respectively) was added and the cells were seeded. A seeding density of 2500 cells/cm^2^ (NHDF) or 3500 cells/cm^2^ (HUVEC) was used for the start of a new culture. After the seeding, the cells were cultured in an incubator (37 °C, 5% CO_2_) and medium was changed every other day. All cells used had passage numbers less than 6.

### Cell attachment

To improve cell adhesion, the membranes were coated with fibronectin solution. Fibronectin solutions of 1 mg/ml, 200 µg/ml and 20 µg/ml were obtained by dissolving fibronectin (Sanquin, Amsterdam) in PBS and then poured on the membranes and incubated for 30 min at 37 °C. After coating, the membranes were incubated with culture medium for 1 h. The cells were seeded (10000 cells/cm^2^) and cultured for 1, 4, and 7 days. The experiment consisted of three groups: a non-coated coverslip as positive control, a non-coated porous PES/PVP membrane as negative control, and a fibronectin coated membrane.

To examine the effect of micropatterned surface topography on the cell attachment, membranes with two different patterns (intermittent lines and combination of intermittent and solid lines) were coated with the optimal concentration of fibronectin and the cells were seeded on top. Non-coated coverslip served as a positive control and fibronectin coated non-patterned membranes served as a negative control. The experiment was performed in triplicate.

In order to visualize cells cultured on non-transparent porous PES/PVP membranes, firstly, cells were fixed in 10% formalin (10 min, room temperature) and washed with PBS (2×). Following fixation, all samples were washed 2× with dH_2_O and stained for 10 seconds with methylene blue (Sigma-Aldrich), after which, they were washed 3× with dH_2_O. Directly after staining, images were taken using a Nikon SMZ800 microscope. Quantification of the amount of cells on the fibronectin coated membranes was done by taking 3 pictures of each membrane and counting the amount of cells.

### Statistical analysis

To determine the effect of fibronectin concentration on cell attachment, a statistical analysis was performed. From all samples (each condition in triplicate), three photos were taken. Since all used cell types have the tendency to grow in clusters after initial attachment, the photos were taken of denser, least dense and average covered areas. The average cell numbers on 1mm^2^ were determined for each sample. Statistical differences in cell numbers between the conditions and control were determined by a Welch’s *T*-test (*P < 0.05; **P < 0.01; ***P < 0.001).

### Cell alignment

To examine the effect of surface topography on cell alignment, the cells were seeded on the top of the micropatterned membranes previously coated with 200 µg/ml fibronectin. The experiment was performed in triplicate both for membranes with intermittent lines as well as ones with combination of intermittent and solid lines. Cells were seeded (10000 cells/cm^2^) and cultured for 7 days, while medium was changed every other day. After culture, the cells were fixed in 10% formalin (10 min, room temperature). After fixation, samples were washed 2× with PBS, permeabilized with 0.05% triton X-100 for 15 min and blocked with 10% goat serum in PBS for 1 h. DAPI was diluted 1:10 in MiliQ water and then 1:10 in PBS and added for 10 minutes to the samples. Images were taken using a BD pathway 435 microscope and analyzed using CellProfiler (v 2.1.1) (Supplementary figure 2). Using this method the nucleus alignment was analyzed. The orientation of the nucleus is defined as the angle between the x-axis with respect to pattern direction and the major axis of the nucleus. All images with cells on micropatterned membranes were aligned horizontally with the x-axis, resulting in a nucleus orientation relative to the patterns. All experiments were performed in triplicate for both cell types. Non-patterned membranes served as a negative control.

### Co-culture

Co-culture experiments of HUVECs together with NHDFs was performed following the protocol provided by Friis et al[34]. NHDFs were first seeded on the membrane with a concentration of 10000 cells/cm^2^ and cultured in FGM. After a confluent layer of NHDFs was obtained, three times as many HUVECs as the starting concentration of NHDFs were seeded on top (30000 cells/cm^2^). The co-culture was kept for 3 days in reduced medium (500 ml EBM-2 supplemented with 0.5 ml ascorbic acid, 2% FBS, 0.5 ml hEGF, 0.5 ml gentamicin sulfate, 0.5 ml heparin, all from the HUVEC media-kit), 1 ng/ml human basic fibroblast growth factor and 10 ng/ml human vascular endothelial growth factor (Preprotech). The experiments were performed in triplicate (for micropatterned and non-patterned membranes) and medium was changed every other day. After 3 days of culture, the cells were fixed in 4% paraformaldehyde (10 min, RT).

### Immunostaining

Samples were washed 2× with PBS and permeabilized with 0.1% triton X-100 (Sigma Aldrich) for 15 min. A 0.1% Tween-20 (Sigma-Aldrich) solution in PBS was made (PBST). The samples were blocked / permeabilized with 10% BSA (Sigma Aldrich) and 22.52 mg/ml glycerin (Sigma-Aldrich) in PBST. Primary and secondary antibody were both diluted in 10% BSA in PBST, 1:200 and 1:400 respectively. Cells were incubated in the diluted primary CD31 antibody (Ab32457, Abcam) for 1 h at RT. After 3 × washing in PBS, the cells were incubated in secondary antibody Alexa 488 (Invitrogen) for 1 h at RT in the dark. After 3 × washing in PBS, cells were counterstained with DAPI (Invitrogen, 1:100 in PBS) for 10 min. Membranes were mounted on cover slides using mounting medium (Hard-set mounting medium, Vectashield). Images were taken using a BD pathway 435 microscope. Autofluorescence from the membrane was manually subtracted from the images by decreasing the range of grey values from the pictures from 0-4095 to 800/1000/1200-4095 (depending on the strength of the autofluorescence and signal) using Fiji software.

### HUVEC structure alignment

The effect of surface topography on the formation of HUVEC structures was studied. Images of HUVECs stained with CD31 on the membranes (three samples of each type of the membrane) were analyzed using the skeletonization plugin in ImageJ (providing region-based shape of the structures). All images with HUVEC structures on micropatterned membranes were aligned horizontally to the x-axis with respect to the pattern direction. The orientation of skeletonized HUVEC structures relative to surface topography (x-axis) was quantified using the OrientationJ plugin.

## Results

### Cell attachment on the membranes

**Fig. 1:**
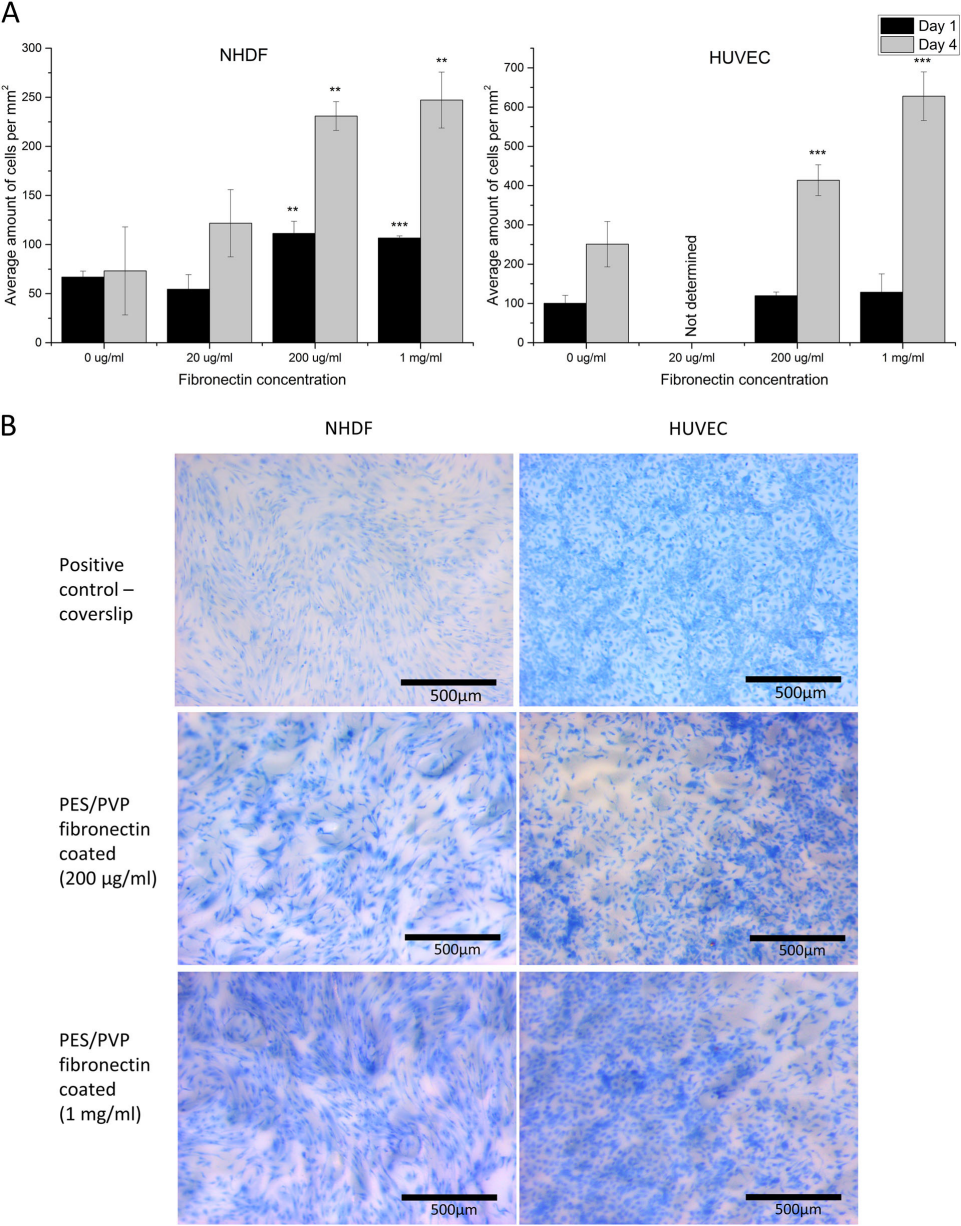
NHDF and HUVEC culture on PES/PVP membranes **a** Average number of NHDF and HUVEC attached per mm^2^ on PES/PVP membranes coated with various fibronectin concentrations. Significance levels: *P < 0.05; **P < 0.01; ***P < 0.001, *n* = 3. **b** Images of methylene blue stained NHDFs and HUVECs cultured for 4 days on coverslip-positive control, non-coated PES/PVP membranes and membranes coated with 200 µg/ml and 1 mg/ml fibronectin

In order to enhance cell attachment to porous PES/PVP membranes, which have low adhesive properties, we applied a fibronectin coating. Fig. 1a compares cell attachment on PES/PVP membranes coated with various fibronectin solutions. After 1 day of culture, a significant increase in NHDF attachment was observed on the membranes coated with 200 µg/ml (*P* < 0.01) and 1 mg/ml (*P* < 0.001) of fibronectin, in comparison to non-coated membranes. Longer culture, for 4 days, confirmed the positive effect of the coating on NHDF attachment. Besides the increase in initial NHDF attachment to the membranes, the fibronectin coating also improved cell proliferation. In fact, all coated membranes performed better after prolonged culture in terms of number of cells per mm^2^, in comparison to non-coated membranes. The coating with fibronectin concentration of 200 µg/ml and 1 mg/ml showed positive results in case of NHDF attachment, therefore, these two concentrations were used for the HUVEC attachment study. Here, although there was no difference in initial HUVEC attachment, we observed improved cell proliferation on coated membranes after 4 days of culture, similar to NHDFs.

Fig. 1b compares images of cells cultured for 4 days on coverslip-positive control, non-coated membranes and membranes coated with fibronectin (200 µg/ml and 1 mg/ml). A similar number of cells is present on the coated membranes and coverslip controls, while the non-coated membranes clearly have low cell attachment properties. Moreover, on the fibronectin coated membranes, cells were spread over the surface and their morphology was preserved comparably to the positive controls. The fibronectin coatings of 1 mg/ml and 200 µg/ml had similar results in terms of cell attachment. We, therefore, selected 200 µg/ml fibronectin concentration for coating of PES/PVP membranes.

### Micropattern membrane characterization

**Fig. 2:**
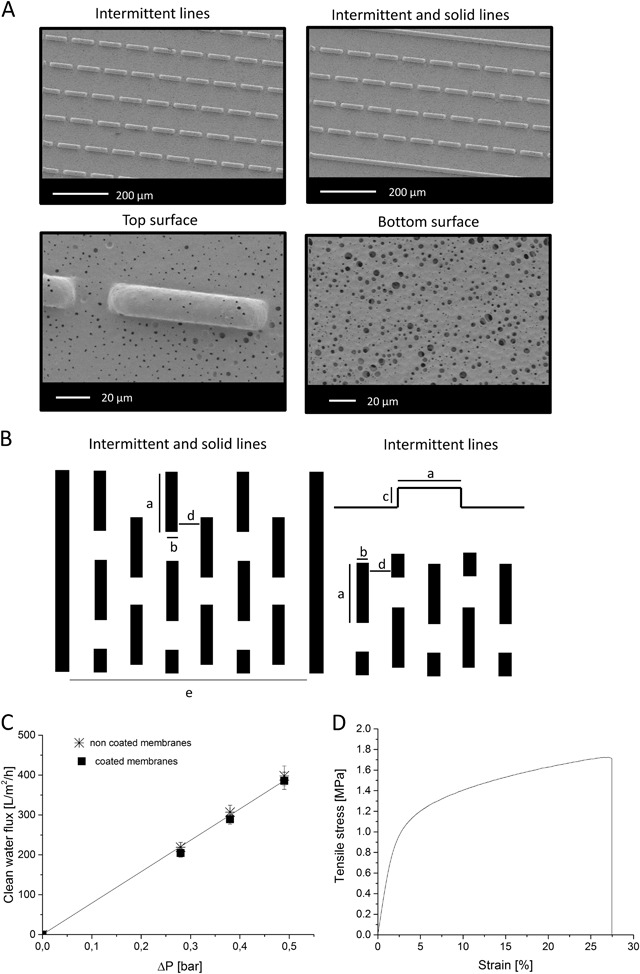
Membrane characteristics **a** Scanning electron microscopy images of micropatterned membranes with intermittent lines and combination of intermittent and solid lines; **b** Schematic representation of patterned on the silicon wafer used for micropatterned membrane fabrication: a = 100, b = 20, c = 40 d = 100, e = 540 µm; **c** Water transport through the patterned PES/PVP membranes; **d** Representative stress-strain curve obtained for PES/PVP membranes

Fig. 2a shows scanning electron microscopy images of micropatterned porous PES/PVP membranes. We obtained high quality micropatterns which closely replicate the designed topography of the silicon wafer used for the membrane fabrication (Fig. 2b). The first micropatterned design consists of equally spaced intermittent lines distributed over the membrane surface, while in the second design, every four rows of intermittent lines are separated by a solid line. The width of intermittent and solid lines is the same as well as the space between each row.

We also observed typical membrane shrinkage during the phase separation process, which helped the release of the micropatterned membrane from the silicon wafer. As a result, we obtained patterns without sharp edges and the distance between the rows of patterns, as well as the length of the intermittent lines, was 20% smaller compared to the designed features on the silicon wafer. The fabricated membranes were porous with the pore size of up to 2 µm on the patterned surface and 1–5 µm pores on the bottom flat surface (obtained from SEM images, see example Fig. 2a). The water transport through the uncoated membranes is identical to the one through coated membranes (water permeability—782 ± 20 L/m2/bar/h, Fig. 2c), proving that the very thin coating does not alter significantly the membrane porosity or pore size. Besides, the graph of the clean water fluxes (CWF) of the membranes at various transmembrane pressures is linear, indicating good mechanical stability of the membranes in the applied pressure range.

Fig. 2d presents a typical shape of stress-strain curve obtained for PES/PVP membranes. The membranes exhibited a Young’s modulus E of 53 ± 2 MPa, a tensile stress at break of 1.75 ± 0.02 MPa and an elongation at break of 27 ± 1%. These values indicate that our membranes are relatively stiff. The contact angle value obtained for PES/PVP membranes was 66 ± 5° confirming membrane moderate hydrophilicity, which increased after coating the membrane with the thin layer of fibronectin (contact angle value of 50 ± 1°).

### Cell alignment on micropatterned membranes

**Fig. 3:**
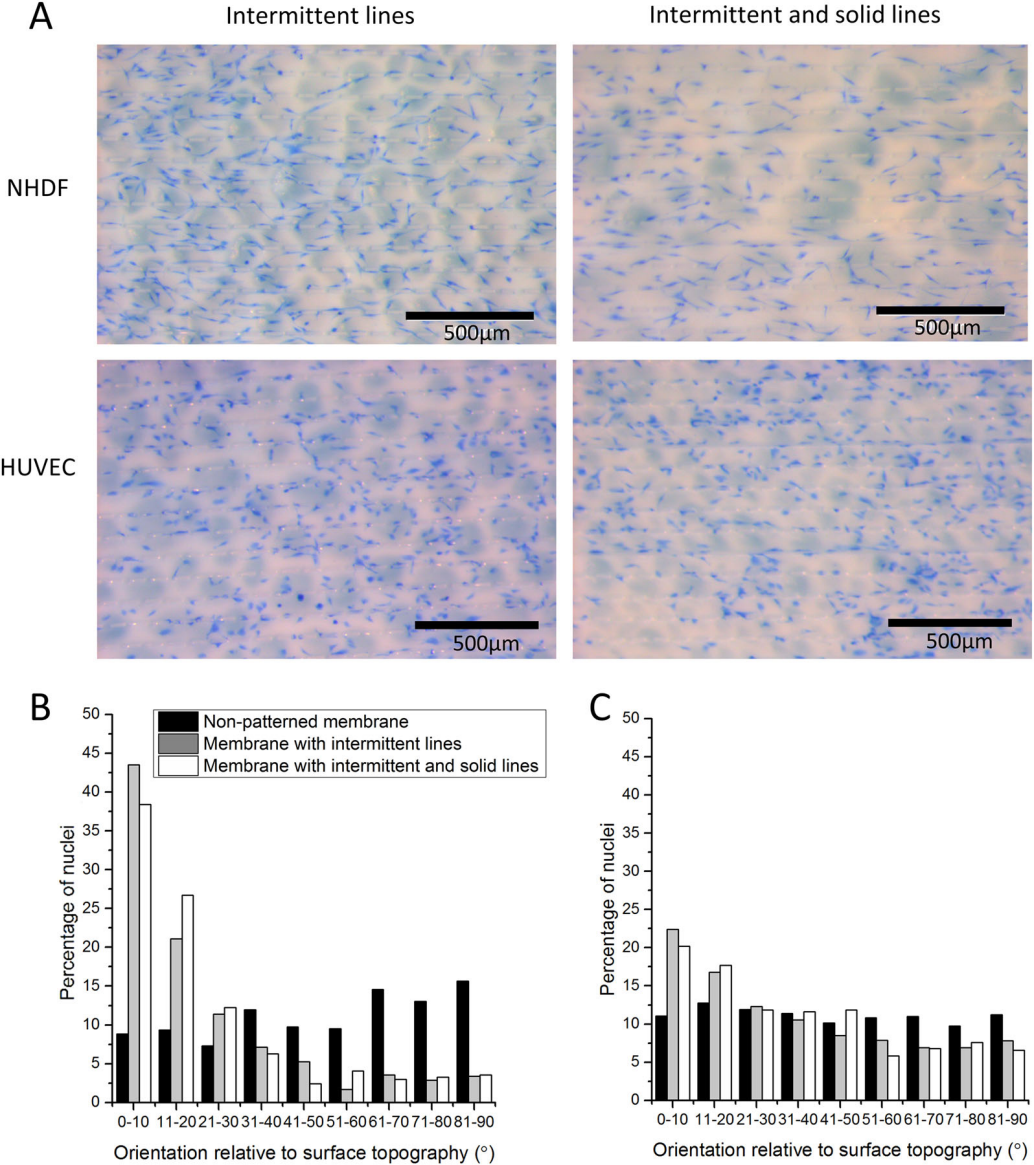
NHDF and HUVEC culture on micropatterned membranes **a** Images of methylene blue stained NHDF and HUVEC after 1 day of culture on micropatterned PES/PVP membranes. Nucleus alignment in relation to the surface topography for **b** NHDFs and **c** HUVECs

Both NHDFs and HUVECs were cultured for 1 and 4 days on micropatterned membranes coated with 200 µg/ml fibronectin solution. Fig. 3a shows images of methylene blue stained NHDFs and HUVECs after 1 day of culture. Initial cell attachment to the micropatterned membranes was similar to the results obtained for non-patterned membranes. Although there are minor differences in cell distribution between membranes with intermittent lines and membranes with combination of intermittent and solid lines (Fig. 3a), after 4 days of culture, all membranes were equally covered with cells (data not shown).

After 1 day, we also observed cell alignment in both NHDF and HUVEC cultures. Figs. 3b, c compares the orientation of cell nuclei on flat and micropatterned PES/PVP membranes, relative to the x-axis of the images with methylene blue stained cells. Both NHDFs and HUVECs cultured on the non-patterned membranes have no particular orientation (similar percent of nuclei for all angles relative to the x-axis of the picture). In contrast, when NHDFs are cultured on a patterned membrane, they show a strong tendency to align to the patterns (angle of 0–20° relative to the microstructures, Fig. 3b), while there are almost no cells growing perpendicular to them. The HUVECs also align to the micropatterns, although the orientation is lower than the NHDFS (Fig. 3c).

### Co-culture of HUVECs and NHDF on micropatterned membranes—effect of surface topography

**Fig. 4:**
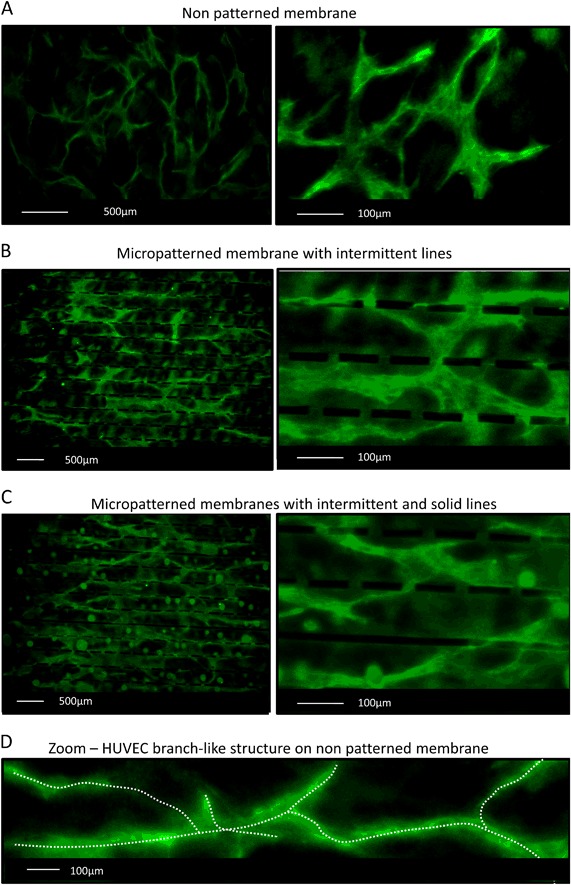
Co-culture of NHDFs and HUVECs resulting in HUVEC network formation. In green the immunostaining for CD31 of HUVEC cells on **a** non-patterned membranes, **b** membrane with intermittent lines, **c** membrane with intermittent and solid lines, **d** example of elongated HUVEC branch-like structure of the network. The dotted line is drawn to guide the eye of the reader

We investigated whether micropatterned membranes have an effect on the HUVEC migration and organization in the subsequent co-culture with NHDFs. HUVECs were seeded on the top of a confluent layer of NHDFs cultured on non-patterned and micropatterned membranes coated with fibronectin. Figs. 4a–c shows CD31 positive HUVECs which form a network on the PES/PVP membranes after 3 days of culture. As expected, HUVECs cultured on the non-patterned membranes have no specific orientation and they migrate and connect, creating elongated branch-like structures within the network in all directions (Fig. 4a, d). The micropatterned membranes, however, show clear cell orientation following the membrane topography (Fig. 4b, c). The membrane patterns guide and assist cell growth during HUVEC network formation. The intermittent lines present on both micropatterned membranes allow for cell interconnection within this network. We also observed that HUVECs sometimes fill the space between the rows of patterns and even grow over the intermittent lines (Fig. 4b—high magnification). As the height of the patterns is the same, cells could also connect over the solid lines, indicating that the addition of this pattern to the intermittent lines still allows for the interconnection between HUVEC branch-like structures within the network (Fig. 4c—high magnification).

**Fig. 5:**
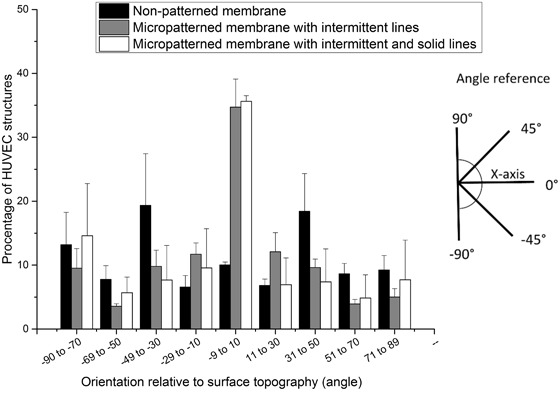
Quantification of HUVEC branch-like structure alignment relative to the x-axis of the immunostaining images (*n* = 3). Membrane patterns were aligned parallel to the x-axis

Fig. 5 compares the percentage of HUVEC branch-like structures within the network on the non-patterned and patterned membranes, for various orientations relative to the x-axis of the immunostaining images. It is clear that cells grow following the direction of intermittent and solid lines forming HUVEC branch-like structures parallel to membrane micropatterns (angle -9°-10° relative to the surface topography), while cells on non-patterned membranes form unorganized HUVEC branch-like structures over the membrane surface. Both micropattern designs lead to the creation of highly interconnected and aligned branch-like structures within the HUVEC network without significant differences between them (Supplementary figure 3).

## Discussion

Islet survival in encapsulation devices is often hindered by lack of adequate vascularization and therefore limited oxygen supply. Vascularization of encapsulation devices often occurs after implantation in an uncontrolled way, triggered by the use of highly porous membranes or addition of stimulating growth factors [2, 7, 10]. Nevertheless, often a period of weeks or months is needed to provide the encapsulated islets with close proximity to functional blood vessels, while hypoxic conditions negatively affect their viability [12]. One of the possible solutions is prevascularization of the device in vitro in order to minimize the time required for the device connection to the host vasculature therefore improving islet survival during the first weeks after implantation [35]. Moreover, it is important to control and guide cell’s organization in order to enhance the microvasculature formation [25].

In our earlier study, we developed a flat macroencapsulation device consisting of a microwell membrane for islet separation and a flat lid membrane. In order for islets to be in close proximity to the blood vessels and avoid harmful hypoxic conditions, we tailored the outer membrane surface topography of our device to induce prevascularization in vitro, allowing for a faster connection with the host vasculature after transplantation. This was achieved by developing PES/PVP micropatterned membranes with surface patterns of intermittent lines and combination of intermittent and solid lines, and we investigated the effect of these patterns on cell alignment and organization, as a first step towards prevascularization. We hypothesized that these micropatterns would assist and guide the cell orientation and therefore would achieve highly organized endothelial cell structures similar to the native tissues. The presence of intermittent lines would allow for the interconnection of the cell network. Since earlier studies showed that cells respond better to topographies at the cell scale, rather than to rough surfaces and topographies above the cell scale (>100 µm), we developed membranes with patterns of 40 µm in height and are 20 µm wide [36–38].

We fabricated PES/PVP micropatterned membranes using phase separation micromolding, which is a unique method for the preparation of porous membranes in one step [30]. The PES/ PVP blend used for membrane fabrication is non-degradable, has good mechanical properties and is widely used as a membrane material for blood purification and other biomedical membrane applications. Moreover, the PES/PVP membranes are relatively stiff (*E* = 53 MPa), which is favorable in terms of cell attachment. In fact, it has been shown that stiffer substrates enhance adhesion of endothelial cells [39]. Cells can sense the stiffness of their substrates through adhesion sites and respond by altering their cytoskeletal structure [40]. Moreover, the PES/PVP membranes are moderately hydrophilic (contact angle of 65°) and have low cell attachment properties. This is favorable for achieving optimal islets transplantation without compromising the insulin delivery. Therefore, in order to improve cell adhesion on one side of the PES/PVP membrane, we applied a thin fibronectin coating which enhanced material hydrophilicity which promotes cell attachment [41]. Fibronectin is a protein of the extracellular matrix (ECM) that, when used as a coating, improves the attachment of the cells by providing more attachment points for the cell focal adhesion points [42]. Indeed, with the fibronectin coating, we achieved an increased attachment of NHDFs and HUVECs on coated membranes in comparison to no-coated ones, similar to coverslip positive controls. The positive effect of fibronectin coating on cell attachment to various materials (e.g., poly (tetrafluoroethylene, PES) was also observed by other researchers [42–44]. Cells are able to attach to ECM molecules such as fibronectin through integrins, activating intracellular signaling pathways directing cell viability, proliferation and differentiation [45]. Therefore, integrin adherence plays an important role in improving cell survival and proliferation. Usually the fibronectin concentration used to enhance cell attachment varies from 20 µg/ml to 1 mg/ml [46–48]. In order to find the optimal coating for PES/PVP membranes, we tested various concentrations of fibronectin. We selected 200 µg/ml fibronectin concentration for coating for our membranes as the coating of 1 mg/ml had similar results in terms of cell attachment. Moreover, the higher coating concentration could result in blocking of membrane pores and transport limitations. Besides an increase in initial cell attachment, the fibronectin coating also improved the proliferation of both NHDFs and HUVECs. Our findings are consistent with the results obtained by Lotz et al. and Pendegrass et.al., who used fibronectin to improve attachment of HUVECs and NHDFs on hydroxyapatite discs and poli(tetrafluoroetylen) films respectively [42, 44]. We also applied a 200 µg/ml fibronectin coating on our micropatterned PES/PVP membranes. As expected, the coating slightly decreased membrane water permeability (6,5%), however, the coating vas very thin and it was not visible on the coated membranes (SEM images similar to non-coated membranes, data not shown).

We further investigated the effect of surface topography on cell alignment. Our results indicate that both cell types, NHDFs and HUVECs, have a tendency to orient and grow along the patterns. Cells guided by both membrane topographies (intermittent lines and combination of intermittent and solid lines) align parallel to the micropatterns, while on the non-patterned membranes they do not show a particular orientation. These findings are consistent with the study of Papenburg et al., where cell alignment was observed on the PLLA membranes with continuous and interconnected channels [30].

Besides the possibility to guide and control the alignment of the single cells, we also investigated whether our surface topographies could affect the formation of endothelial cell organized networks in a co-culture system. The co-culture has been found to closely mimic the in vivo situation and to form stable endothelial cell networks [21]. Therefore, we adapted a protocol from Friis et al. for the co-culture of HUVECs and NHDFs, where the NHDF monolayer cultured on our membranes served as a support for HUVEC network formation [34]. By using this co-culture protocol, we successfully obtained HUVEC network formation on PES/PVP membranes after 3 days of culture, without additional application of hydrogels (e.g., Matrigel), often used as angiogenesis assays [21]. The use of gels could result in blocking of membrane pores and severely hinder membrane transport properties. The endothelial cells, co-cultured on the monolayer of fibroblasts, grew and connected on the surface of non-patterned PES/PVP membranes forming HUVEC networks without specific orientation, similar to the ones obtained on polystyrene surfaces by Friis et al. [34]. Fuchs et al. have also obtained similar HUVEC networks on polycaprolactone disks using co-culture of endothelial cells and primary osteoblasts [49]. Although our results present only preliminary endothelial cell network formation, it has been shown that prolonged co-culture of endothelial cells and fibroblasts can result in capillary lumen formation [34]. Moreover, it has been reported that prevascularized engineered tissue constructs using a co-culture of HUVECs and fibroblasts implanted subcutaneously in immune-deficient mice anastomosed with the host vasculature within 4–5 days after implantation and the rate of vascularization was faster in the prevascularized construct compared to the non-prevascularized one [50]. In our study, we focused on the possible guidance and interconnectivity of the HUVEC networks in order to mimic closely the highly organized native tissues, as a first step towards the prevascularization of our encapsulation device in vitro. Importantly, the intermittent line pattern allowed cell communication and interconnection between the cells growing in parallel rows of patterns. In other studies, aligned and elongated HUVEC structures were obtained using grooves, stripes or adjacent fibers to affect HUVEC orientation [26–28]. Nevertheless, these methods did not allow for the connection between the structures, which is important the formation of the microvascular network. The advantage of our micropattern design is that, besides assisting cell alignment, the intermittent lines allow for the interconnection of HUVEC branch-like structures, forming an endothelial cell network. In case of both membranes (either with intermittent lines or with solid and intermittent lines) we observed a similar effect on HUVEC branch-like structures organization. The addition of solid to intermittent lines did not block the connections between forming HUVEC branch-like structures. We observed that HUVECs connected over the solid lines, indicating that the line dimensions (40 µm in height and 20 µm wide) allowed for signaling and communication between the cells.

## Conclusion

In this study, we established the co-culture of HUVECs and fibroblasts grown without addition of hydrogels on micropatterned PES/PVP membranes, coated with a thin layer of fibronectin. By using these membranes with micropatterns of intermittent lines, as well as, their combination with solid lines, we achieved interconnected HUVEC branch-like structures oriented in the direction of the patterns, which is an important step towards obtaining a stable endothelial cell network for the prevascularization of our flat encapsulation device.

In the future, we will investigate the application the micropatterned membrane as a lid to the microwell encapsulation device [24]. The micropatterned surface would support cell organization during the development of a prevascularized layer on the outside of the device. As the thickness of the membrane can be easily tailored during membrane fabrication, we can minimize the distance between encapsulated cells and of the prevascularized surface to less than 200 µm, which correlates with the distance for achieving appropriate oxygen delivery to the cells [2]. After the organized microvessel-like structures are established in vitro, islets can be seeded inside the device and transplanted. The presence of a highly interconnected prevascularized layer, closely mimicking native tissue, could reduce the time of construct reconnection to host vasculature and improve islet survival within our macroencapsulation device.

In this study, we used human umbilical vein derived endothelial cells as they have been employed in many studies as an endothelial cell model for experiments attempting to achieve microvessel formation and vascular remodeling [51–53]. Moreover, HUVECs are easily extracted from an available supply of discarded umbilical cords and can be expanded to relatively large numbers. However, in order to minimize alloimmune mechanisms leading to implant rejection, the use of autologous fibroblasts and endothelial cells needs to be considered in the case of clinical application of our strategy. The fibroblasts can be relatively easily obtained from subcutaneous tissue while allogeneic HUVECs could be replaced with an autologous endothelial cell source such as endothelial colony–forming cells (ECFCs) isolated from human peripheral blood or adipose tissue endothelial cells (AT-ECs) [54, 55].

## Electronic supplementary material

Supplementary figures
