# Supplementary material for: An important step towards a prevascularized islet macroencapsulation device—effect of micropatterned membranes on development of endothelial cell network
Source: J Mater Sci Mater Med. 2018 Jun 25;29(7):91. doi: 10.1007/s10856-018-6102-0 (PMC6018599; doi:10.1007/s10856-018-6102-0)
Supplement: Supplementary file 1 — Supplementary figures [file 10856_2018_6102_MOESM1_ESM.docx]

**An important step towards a prevascularized islet macroencapsulation device - Effect of micropatterned membranes on development of endothelial cell network**

Katarzyna Skrzypek^a^, Milou Groot Nibbelink^b^, Lisanne P. Karbaat^b^, Marcel Karperien^b^, Aart van Apeldoorn^b,c^, Dimitrios Stamatialis^a^

Author affiliation

^a^Bioartificial organs, Biomaterials Science and Technology, MIRA Institute of Biomedical Technology and Technical Medicine, University of Twente, The Netherlands

^b^Developmental BioEngineering, MIRA Institute of Biomedical Technology and Technical Medicine, University of Twente, The Netherlands

^c^Present address: Complex Tissue Regeneration, MERLN Institute for Technology Inspired Regenerative Medicine, Maastricht University, The Netherlands

**Corresponding author:**

**Dimitrios Stamatialis**

**d.stamatialis@utwente.nl**

**P.O Box 217, 7500 AE Enschede, The Netherlands**

**tel: +31534894675**


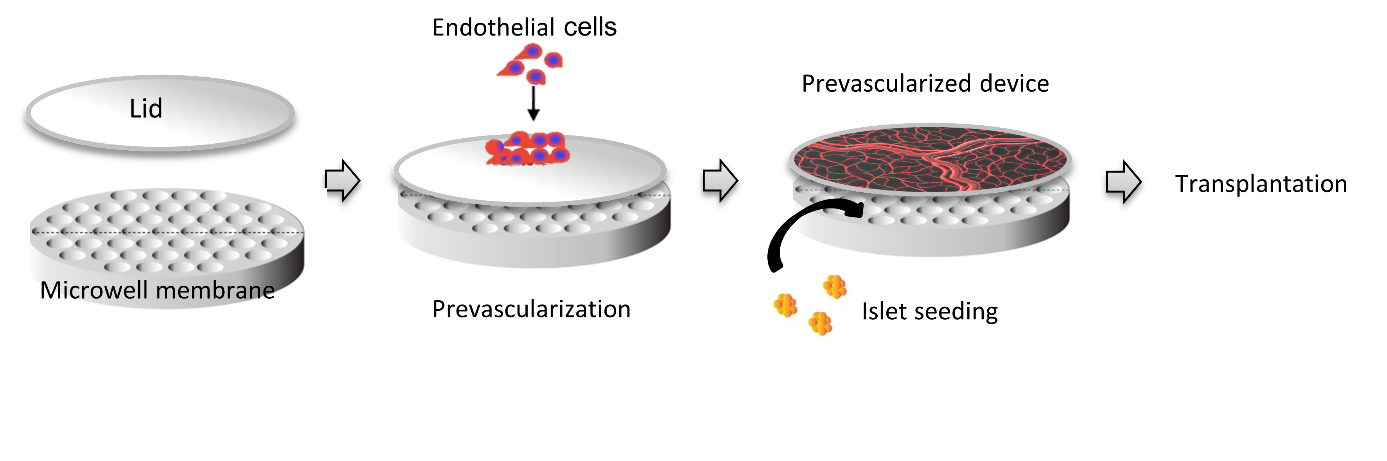


***Supplementary figure 1. Schematic representation of the final flat macroencapsulation device consisting of two PES/PVP membranes: microwell membrane for islets separation inside the device and covering membrane designed for prevascularization of the device.***


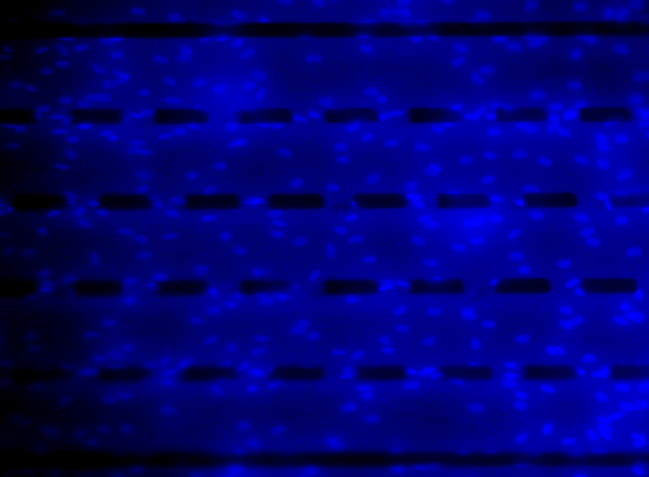


***Supplementary figure 2.*** *Example of an image with cell nuclei stained with DAPI on the membranes with intermittent and solid lines used in CellProfiler to determine cell alignment.*


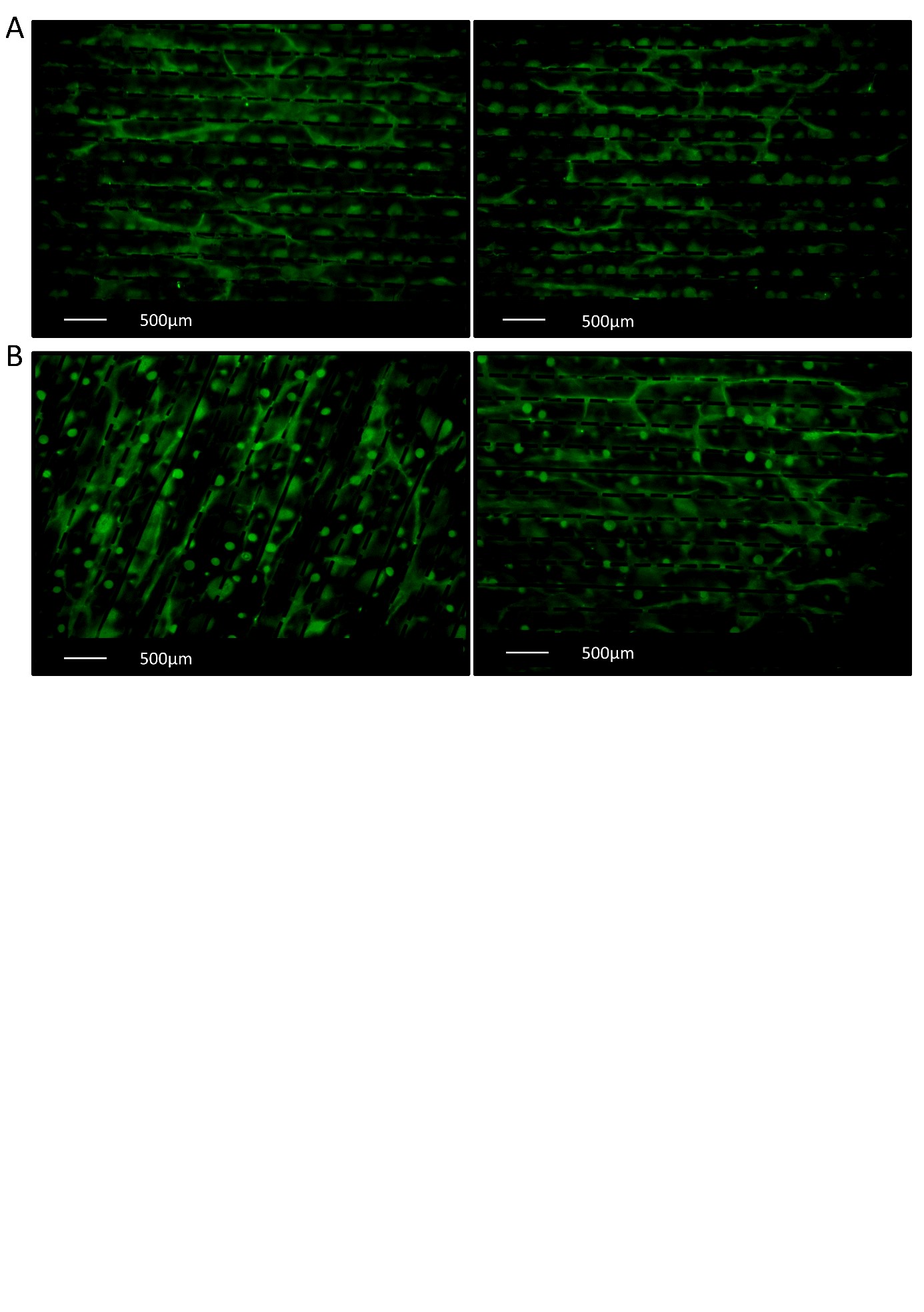


***Supplementary figure 3. Co-culture of NHDFs and HUVECs resulting in HUVEC network formation. In green the immunostaining for CD31 of HUVEC cells on A) membranes with bricks, B) membranes with bricks and channels.***
